# Cytokinin-Regulated Expression of *Arabidopsis thaliana PAP* Genes and Its Implication for the Expression of Chloroplast-Encoded Genes

**DOI:** 10.3390/biom10121658

**Published:** 2020-12-11

**Authors:** Aleksandra A. Andreeva, Radomira Vankova, Ivan A. Bychkov, Natalia V. Kudryakova, Maria N. Danilova, Jozef Lacek, Elena S. Pojidaeva, Victor V. Kusnetsov

**Affiliations:** 1 Timiryazev Institute of Plant Physiology, Russian Academy of Sciences, Botanicheskaya 35, 127276 Moscow, Russia; Alexaa27@mail.ru (A.A.A.); ivan.a.b@mail.ru (I.A.B.); mariadanilova86@yandex.ru (M.N.D.); alenapoj@mail.ru (E.S.P.); vkusnetsov2001@mail.ru (V.V.K.); 2 Institute of Experimental Botany, Czech Academy of Sciences, Rozvojova 263, 165 02 Prague, Czech Republic; vankova@ueb.cas.cz (R.V.); lacek@ueb.cas.cz (J.L.)

**Keywords:** *Arabidopsis thaliana*, chloroplast biogenesis, cytokinin, PEP-associated proteins, plastid transcription machinery

## Abstract

Cytokinins (CKs) are known to regulate the biogenesis of chloroplasts under changing environmental conditions and at different stages of plant ontogenesis. However, the underlying mechanisms are still poorly understood. Apparently, the mechanisms can be duplicated in several ways, including the influence of nuclear genes that determine the expression of plastome through the two-component CK regulatory circuit. In this study, we evaluated the role of cytokinins and CK signaling pathway on the expression of nuclear genes for plastid RNA polymerase-associated proteins (PAPs). Cytokinin induced the expression of all twelve *Arabidopsis thaliana* *PAP* genes irrespective of their functions via canonical CK signaling pathway but this regulation might be indirect taking into consideration their different functions and versatile structure of promoter regions. The disruption of *PAP* genes contributed to the abolishment of positive CK effect on the accumulation of the chloroplast gene transcripts and transcripts of the nuclear genes for plastid transcription machinery as can be judged from the analysis of *pap1* and *pap6* mutants. However, the CK regulatory circuit in the mutants remained practically unperturbed. Knock-out of PAP genes resulted in cytokinin overproduction as a consequence of the strong up-regulation of the genes for CK synthesis.

## 1. Introduction

Chloroplast development and function requires the coordinated transcriptional regulation of genes encoded by the nuclear and chloroplast genomes. Phytohormones, in particular cytokinins (CK), are among the main factors affecting differential expression of the genes encoding plastid or plastid-related proteins encoded by the nucleus. Genome-wide transcript analyses revealed in *Arabidopsis thaliana* more than 100 cytokinin-responsive genes related to photosynthesis, in addition to a list of genes implicated in cytokinin-regulated chlorophyll biosynthesis and the expression of plastid genes [1,2,3].

Cytokinins act on plastids via a multistep signaling system consisting of histidine kinases, histidine phosphotransfer proteins (AHP) and type-A and type-B response regulators (RRs). In parallel, the system includes a subset of cytokinin response factors (CRFs) which are induced either directly by phosphorylated AHPs or by type-B RRs. The activated type-B RRs together with CRFs mediate CK-regulated gene expression through directly binding to specific sites on the promoter regions of CK-regulated genes [4].

Among the multiple target genes of cytokinin action is HY5, a basic leucine zipper transcription factor that functions downstream of the light receptor network, and two transcription factor genes GNC (GATA, nitrate inducible, carbon metabolism involved) and GNL (GNC-like, also known as cytokinin responsive GATA factor 1, CGA1) belonging to plant type IV zinc finger proteins. These genes are considered as key regulators of a transcriptional network integrating both light and cytokinin effects on chloroplast biogenesis [5,6]. Overexpression of *GNC* and *GNL* facilitates differentiation of proplastids into chloroplasts in the light and to etioplasts in the dark and promotes greening in roots [7]. Both genes act at the expression level, in an additive manner with the CK-induced *GLK1* (*Golden-like1*) and *GLK2* (*Golden-like2*) transcription factor genes which are implicated in chloroplast development and photosynthetic activity. However, plastome-encoded genes cannot be direct targets of the GNC or GNL families of transcription factors, despite a substantial decrease of their expression rates in *gnc cga1* and *glk1 glk2* mutants [6]. Therefore, the observed effect should be interpreted as indirect, resulting from the regulation of nuclear gene expression by GNC or GNL transcription factors.

A diverse array of the CK-dependent nuclear genes that are critical for chloroplast biogenesis comprises the genes of chloroplast transcriptional complex. Plastid genes are transcribed by two principally different transcription systems including nuclear encoded phage type RNA polymerases (NEPs) and plastid-encoded eubacterial type RNA polymerase (PEP). NEPs are predominantly involved in transcription of housekeeping genes at early developmental stages, though in PEP-deficient and other albino mutants NEPs were shown to transcribe even photosynthesis genes from remote promoters [8,9]. PEP is mainly responsible for transcription of photosynthesis genes. It exists in two forms: PEP-B composed of 4-plastid encoded core subunits (α, β, β’ and β’’) and a sigma factor and PEP-A where core subunits of multimeric PEP became decorated with at least 12 additional nuclear encoded proteins [9,10]. PEP-B is responsible for the activity in etioplasts and at the early stage of greening, while PEP-A becomes active during light-dependent chloroplast development [11]. We have shown that CK treatment induced the expression of the nuclear-encoded *RPOTp*, *RPOTmp*, as well as of *SIG1*, *2* and *6*, and down-regulated *SIG4* and *5* in AHK2/AHK3-dependent manner [12]. A direct proof of GNL-dependent regulation of *SIG* family transcription factor genes was recently presented by Bastakis et al. [13]. According to their ChiP-qPCR experiments, promoter elements of *SIG2* and *SIG6* transcription factor genes containing GATA sites were bound by GNL:HA and this GATA-dependent regulation could be extended to four other SIG family members. Therefore, a reduced response of chloroplast genes to cytokinin in *sig1, 2* and *6* mutants revealed by Borcellino [14] might be explained by the involvement of sigma factors in CK-dependent fine tuning of the expression of plastid genes.

PEP-associated proteins (PAPs) essential for the proper assembly and/or stability of the PEP complex [9] are subdivided into three major groups according to their predicted functional domains [15]. Group one (PAP1, 2, 3, 5 and 7) is implicated in DNA/RNA metabolism, group two is engaged in redox-related functions (PAP6 and 10) and ROS protection (PAP4 and 9). Group three comprises proteins with unknown or metabolism related functions (PAP8, 11 and 12) [15]. Despite the fact that PAPs are highly diverse in their structure and function, inactivation of their corresponding genes causes the same phenotypic consequences (albino or pale green plants). In co-expression analysis the *PAP* genes show a high degree of correlation in transcript accumulation suggesting that they might represent a regulon [9]. At the same time *PAP* gene expression demonstrates a high tissue specificity and developmental progression coupled with a selective impact of light [15]. This suggests that *PAP* gene expression may be controlled by interplay between light and hormone-dependent signaling pathways superimposed on internal developmental signals.

In this study, we have made attempt to clarify the role of cytokinins in the regulation of *PAP* gene expression and the possible outcome for the expression of genes encoded by organelles. Our data revealed that *PAP* genes are involved in CK-mediated regulation of the chloroplast transcription machinery via canonical CK signaling pathway but disruption of *PAP* genes promotes hormone overproduction and the transition of CK-dependent accumulation of chloroplast encoded proteins and their mRNA levels from a positive to a negative one.

## 2. Materials and Methods 

### 2.1. Plant Material, Growth Conditions, and Cytokinin Treatment

*Arabidopsis thaliana* L ecotype Col-0 seeds were stratified and then germinated either in soil or in Murashige and Skoog (MS) medium that contained 2% sucrose. Seedlings were grown in controlled-environment chamber for indicated periods up to the age of 7 weeks under a 16 h light/8 h dark cycle at 23 °C and 100 µE m^−2^ s^−1^ light intensity. For CK treatment plants were sprayed with a solution containing 5 μM *trans*-zeatin or an equal aliquot of ethanol. The duration of the treatment varied from 1 to 24 h before harvest. To average the results, leaves from at least six different plants for each probe were collected. T-DNA insertion mutants (all are ecotype Col-0) were obtained from the Nottingham Arabidopsis Stock Centre or kindly provided by Prof. Tatsuo Kakimoto from Osaka University, Japan. Homozygosity of the mutant lines was verified by PCR genotyping. In addition, the lack of expression of the knockout genes was further confirmed by real-time PCR.

### 2.2. Hypocotyl and Root Elongation Assays

For hypocotyl measurements, seedlings were grown on vertical plates in a medium supplemented with 10^−7^, 10^−6^ and 10^−5^ M *trans*-zeatin for 4 days in the dark. Homozygous *pap1* and *pap6* hypocotyls were identified based on white phenotype of cotyledons after cultivating for additional 24 h under lights. For root elongation assay, seedlings were grown in white light for 6 days on vertical plates containing MS with a range of *trans*-zeatin concentrations and the length of the primary root was determined.

### 2.3. Protein Retention Assay

Seedlings were grown in vitro on horizontal plates for 5 weeks. Leaves from approximately the 3nd and the 4th layers (from the 5th to the 8th true leaves) were detached and incubated in the dark at 23 °C on water or *trans*-zeatin solution (5 × 10^−6^ M) for 3 days. Total soluble protein was determined by the bicinchoninic acid (BCA) method using Pierce BCA assay kit (Thermo Fisher Scientific, Rockford, Illinois, USA) and expressed µg per cm^2^.

### 2.4. Analysis of Transcript Levels by Quantitative Real Time (RT)-PCR

The expression levels of nuclear and chloroplast genes were evaluated by qRT-PCR according to Danilova et al. [12] in a LightCycler 96 (Roche diagnostics international ltd., Roche, Switzerland) with minor modifications. The following standard thermal profile was used for all PCR reactions: 95 °C for 5 min, 40 cycles of 95 °C for 15 s, 58 °C 15 s and 72 °C for 25 s. Primers are listed in the Supporting Information section (Table S1). All data were normalized to the amount of the transcript levels of the nuclear encoded *UBQ10* (*At5g53300*) or protein phosphatase 2A (PP2A) regulatory subunit A2 (*At3g25800*) genes which were used as internal control.

### 2.5. Protein Isolation and Western Blot Analysis

Total protein extracts were prepared from the homogenized leaf material in a buffer containing 10 mM Tris-HCl, pH 8.0, 0.5% SDS, 4% glycerol, and 0.1 mM EDTA, at pH 8.0. After homogenization the samples were refrozen in liquid nitrogen and subjected to mild ultrasonic treatment (pulse of 15 s, amplitude of 30%) until slight thawing. The cycle was repeated 3 times. Protein concentrations were determined by the BCA method using the Pierce BCA protein assay kit (Thermo Fisher Scientific, Rockford, IL, USA). Ten to twenty micrograms of total protein were separated by 10% SDS-PAGE NEXT GEL (Amresco, Solon, OH, USA) and transferred to nitrocellulose membrane. Membranes were blocked with 2–5% of low-fat milk (Sigma Aldrich, Sant Louis, MO, USA). They were then incubated with anti-PsbD (PSII; AS06 146), anti-RbcL (Rubisco; AS03 037), anti-PsaB (PSI; (AS10 695)), anti-Lhcb2 (AS01 003), anti-AtpB (β subunit of ATP synthase complex; AS05 085), anti- AccD (acetyl-CoA carboxylase subunit; AS15 2880) and anti-RpoB (RNA polymerase beta subunit (chloroplast); AS15 2867) primary antibodies (Agrisera, Vännäs, Sweden) overnight at 4 °C followed by the secondary antibody (anti-rabbit IgG horseradish peroxidases conjugated from Agrisera, ASO9 602) for 1 h at room temperature according to the manufacturer’s instructions. Signals from immunoblotting were detected using the ECL method (ECL western blotting detection kit, Bio-Rad, Hercules, CA, USA) by Li-Cor western blot imager (Li-Cor, Lincoln, NE, USA). Representative results are shown. Western experiments were performed in three replicates. Figures present typical results.

### 2.6. Hormone Extraction, Purification, and Determination

Frozen samples (ca 10 mg FW) were homogenized and extracted with cold (−20 °C) methanol/water/formic acid (15/4/1, *v*/*v*/*v*) as described in Dobrev and Kaminek [16] and Dobrev and Vankova [17]. The following isotope-labelled internal standards (10 pmol/sample) were then added ^2^H_5_-tZ, ^2^H_5_-tZR, ^2^H_5_-tZRMP, ^2^H_5_-tZ7G, ^2^H_5_-tZ9G, ^2^H_5_-tZOG, ^2^H_5_-tZROG, ^2^H_3_-DZ, ^2^H_3_-DZR, ^2^H_3_-DZ9G, ^2^H_3_-DZRMP, ^2^H_7_-DZOG, ^2^H_6_-iP, ^2^H_6_-iPR, ^2^H_6_-iP7G, ^2^H_6_-iP9G, ^2^H_6_-iPRMP (Olchemim, CR, Olomouc, Czech Republic). Phytohormones were separated with a reverse phase-cation exchange SPE column (Oasis-MCX, Waters, Milford, MA, USA) into the acid fraction by elution with methanol and into the basic fraction by elution with 0.35 M NH_4_OH in 60% methanol which was used for CK determination. The latter fraction was analyzed using HPLC (Ultimate 3000, Dionex, Sunnyvale, CA, USA) coupled to 3200 Q TRAP hybrid triple quadrupole/linear ion trap mass spectrometer (Applied Biosystems, Waltham, MA, USA). Hormone quantification was performed by the isotope dilution method with multilevel calibration curves (r^2^ > 0.99). Data processing was performed with the Analyst 1.5 software package (Applied Biosystems, Waltham, MA, USA).

### 2.7. Statistical Data Processing

The experiments were performed in three biological replicates and the results were averaged. All data are presented as the mean values ± their standard errors (SE). The means were compared using a Student’s *t*-test.

## 3. Results

### 3.1. The Expression of PAP Genes Is Regulated by Cytokinin and Requires the Elements of CK Signaling Pathway

To identify *PAP* genes potentially regulated by CK, their promoter sequences were examined for the occurrence of CK-related *cis*-elements. For this purpose, approximately 500 bp sequences located upstream of the translation start sites (TSS) and 50 bp downstream of the TSS were analyzed for the putative CK-regulated targets using AthaMap server http://www.athamap.de/ [18]. We found that the promoters of all *PAP* genes tested contained at least several ARR-B binding motifs and *GLK1* binding sites, in addition to some extra motifs which were present only in a few promoters (Table S2). Therefore, regulation of *PAP* genes can be mediated by direct transcriptional factor-DNA binding interactions. 

Cytokinin responsiveness is tightly linked with the physiological state of plants and ontogenetic programs that determine the specific time windows for the implementation of hormonal signals. Therefore, we examined the expression of *PAP* genes in response to *trans*-zeatin (5 μM) after 3 h of treatment at three developmental stages by qRT PCR analysis. The duration of CK treatment was chosen after preliminary tests. The analyses were performed in the entire 7-day-old seedlings, and the 6th true rosette leaf harvested from 3- and 7-week-old wild type (Col-0) plants. For each gene relative expression values were normalized to the transcript levels of 7-day-old seedlings. The nuclear gene *ARR5* (for a type-A negative regulator) was used as a marker of CK action. 

**Figure 1:**
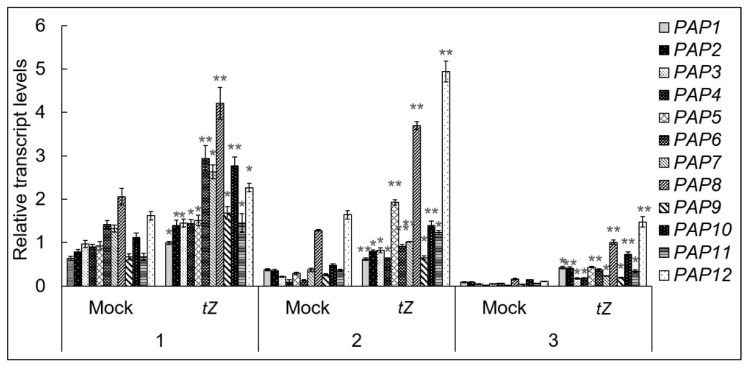
Age-related changes in the accumulation of *PAP* gene transcripts after trans-zeatin (5 μM, 3 h) treatment. *A. thaliana* Col-0 plants were grown on MS medium in Petri dishes for one week or in soil under a 16 h light/8 h dark photoperiod at 23 °C with 100 μE m^−2^ s^−1^. Total RNAs were isolated from 7-day-old seedlings (1) or the 6th leaf of 3-(2) and 7-(3) week-old *A. thaliana* plants. RNAs were analyzed by relative quantitative RT-PCR using *UBQ10* and *PP2A* as internal standards. Data represent the means and SEs values of three independent biological replicates. Asterisks indicate significant differences from the value of respective mock control samples (* *p* < 0.05; ** *p* < 0.01).

The comparison of all three stages of plant development showed that the strongest CK induced changes in transcripts of PAP genes occurred in 7-week-old plants. Exogenous CK had a robust effect due to low endogenous CK levels, promoting considerably the signal transduction (Figure 1). Simultaneously, steady state levels of all *PAP* mRNAs were maximal in cotyledons of 7-day-old seedlings. The results revealed a general increase of all *PAP* transcripts irrespective of their functions and the stage of plant development by CK. The induction by exogenous CK was less pronounced in 7-day-old seedlings, which have high levels of endogenous CKs and thus total active CK increase was relatively lower.

**Figure 2:**
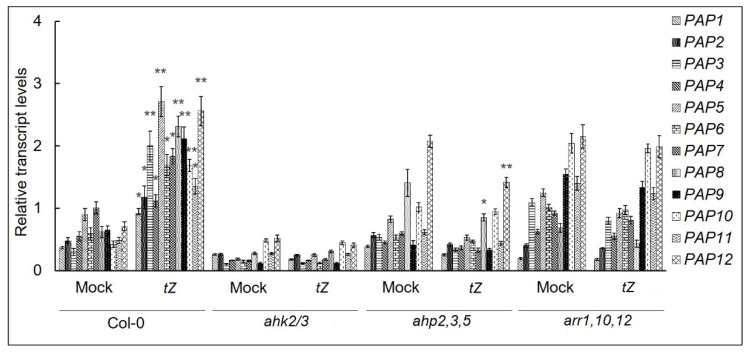
Effects of *trans*-zeatin (5 μM, 3 h) on the transcript accumulation of *PAP* genes in 1-week-old wild type plants and CK signaling mutants. *A. thaliana* Col-0 and mutant plants were grown on MS medium in Petri dishes for 7 days under a 16 h light/8 h dark photoperiod at 23 °C with 100 μE m^−2^ s^−1^. Total RNAs were isolated from seedlings and analyzed by relative quantitative RT-PCR using *UBQ10* and *PP2A* as internal standards. Data represent the means and SEs values of three independent biological replicates. Asterisks indicate significant differences from the value of the respective mock control samples (* *p* < 0.05; ** *p* < 0.01).

In order to elucidate which elements of CK signaling pathway mediates the effects of CKs on the accumulation of *PAP* transcripts, the loss-of-function approach was used to evaluate the dependence of *PAP* gene expression on the cytokinin receptors AHK2 and AHK3. Double mutant *ahk2,3* did not display elevation of *PAP* mRNA levels after CK treatment (Figure 2), in contrast to Col-0 control. Likewise, disruption of the genes for phosphotransfer proteins AHP 2, 3 and 5 (i.e., the *ahp2,3.5* mutant) and type-B ARR1, 10 and 12 (the *arr1,10,12* mutant) reduced the response of *PAP* genes to CK. Thus, cytokinin-regulated expression of *PAP* genes requires the activity of the canonical cytokinin signaling pathway.

### 3.2. Loss of Function pap1 and pap6 Mutants Exhibited an Altered Response to Cytokinin Treatment

According to the generally accepted paradigm, the key role in the identification and confirmation of the effect of a gene and of its possible physiological function is played by the knockout mutants with the disrupted function of the coding gene. To further substantiate that PAPs are actually regulated by CK, the *Arabidopsis thaliana pap1/ptac3* insertion line (NASC 639260) was analyzed. PAP1 is a typical PAP protein with a predicted size of 110 kDa, which was shown to be associated with the PEP complex at all three steps of the transcription cycle including initiation, elongation and termination [11]. It contains a SAP DNA-binding domain, and PPR motif involved in RNA metabolism, in addition to a domain associated with the stabilization of *rbcL* transcripts [9]. Yagi et al. [19] proposed that PAP1 is a PEP-associated general, rather than sequence-specific, transcription factor since it binds in a light dependent manner to the transcribed regions of all PEP-dependent photosynthesis and rRNA related genes tested. In silico examination of 5′ region of *PAP1* revealed the presence of type-B ARR consensus binding sites (A/G)GAT) suggesting the possibility that *PAP1* may be a CK-dependent target gene. In addition, it contained binding elements for GLK1, HY5 and GATA 1,2,3,4 and 12 transcription factors consistent with the notion that *PAP1* could be their direct target (Table S2).

As a further independent control, a mutant disrupted for *pap6/fln1* (NASC 305140) was also tested. *PAP6* codes a protein of 49 kDa that contains a phosphofructokinase domain, though a fructokinase activity of the protein has not been confirmed. The protein has been shown to interact with thioredoxin domain of PAP10/Trx and may act as part of the signaling chain involved in the redox regulation of PEP function in chloroplasts [9,20]. *PAP6* is up-regulated at elevated temperatures [21] similarly to *OsFLN2*, which has been shown to encode the putative fructokinase-like protein, acting as part of the PEP complex in rice [22].

**Figure 3:**
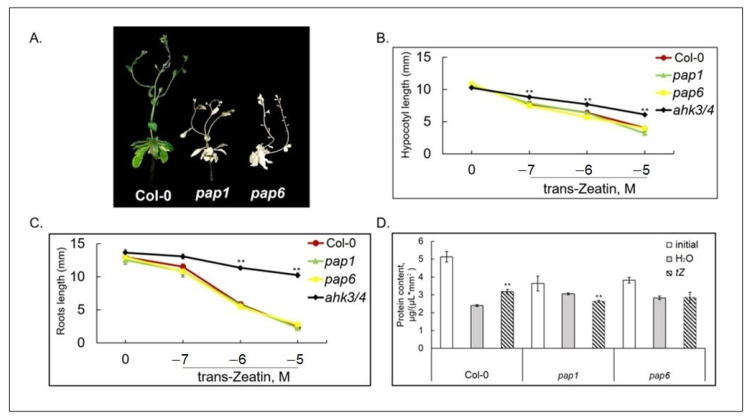
CK responses of *A. thaliana* Col-0 plants and pap mutants: (**A**) 6-week-old plant grown on MS medium under a 16 h light/8 h dark photoperiod at 23 °C with 100 μE m^−2^ s^−1^. Inhibition of hypocotyl (**B**) and root (**C**) elongation by trans-zeatin. For hypocotyl measurements seedlings were grown on vertical plates in a medium supplemented with 10^−7^, 10^−6^ and 10^−5^ M *trans*-zeatin for 4 days in the dark. For root elongation assay, seedlings were grown in white light for 6 days on vertical plates containing MS with a range of *trans*-zeatin concentrations. The *ahk3/4* mutant was used as a negative control. Asterisks indicate significant differences from the Col-0 (* *p* < 0.05; ** *p* < 0.01). (**D**) Sensitivity to CK during dark-induced senescence. Total protein content was measured in detached leaves (3nd and the 4th layers) of 5-week-old *A. thaliana* Col-0 plants and *pap* mutants incubated in the dark at 23 °C on water or *trans*-zeatin solution (5 × 10^−6^ M) for 3 days. Asterisks indicate significant differences from respective mock controls.

Both homozygous mutants, *pap1/ptac3* and *pap6/fln1* exhibited albino phenotypes viable only on sucrose supplemented medium, displaying severe defects in chloroplast development (Figure 3A). The response of the mutant seedlings to exogenous CK was initially evaluated by root and shoot elongation assays. The mutants responded to CK treatment similarly to wild type seedlings, confirming unchanged sensitivity to the hormone (Figure 3B,C) at early developmental stages. However, during senescence of detached leaves, protein degradation was not retarded by CK in *pap6* mutant as in wild type plants, while in CK-treated *pap1* it occurred even more rapidly than in the water control (Figure 3D). Thus, CK-mediated ability to retard senescence is compromised in both *pap* mutants.

**Figure 4:**
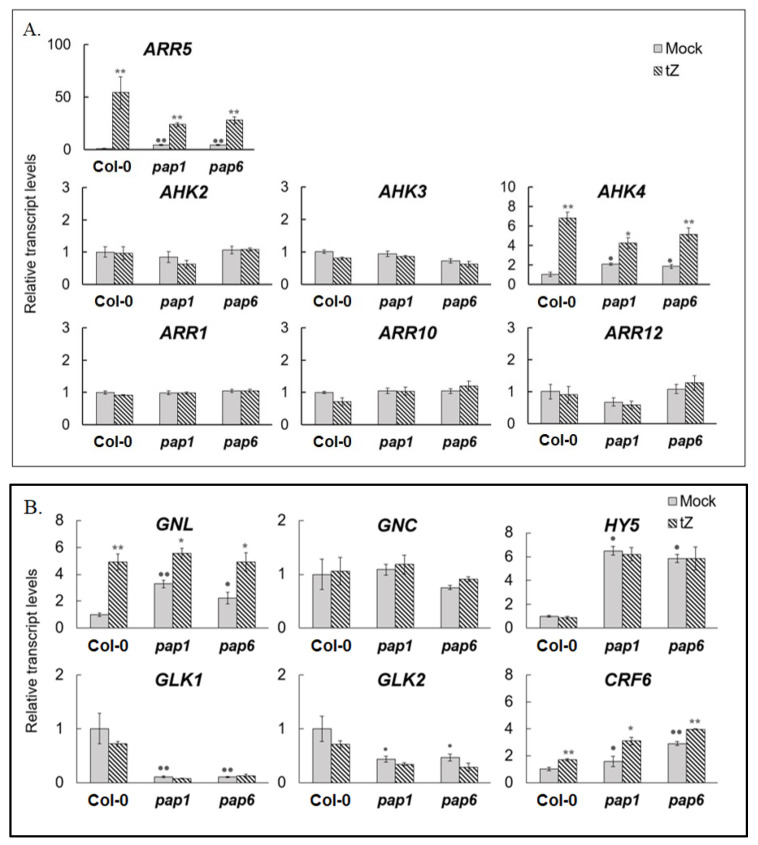
Relative expression values of *A. thaliana* CK signaling genes (**A**,**B**) in untreated 4-week-old wild type plants and *pap* mutants, and under CK (*trans*-zeatin 5 μM, 3 h) treatment. *A. thaliana* Col-0 and mutant plants were grown on MS medium in Petri dishes for four weeks under a 16 h light/8 h dark photoperiod at 23 °C with 100 μE m^−2^ s^−1^. Total RNAs were isolated from rosette leaves and analyzed by relative quantitative RT-PCR using *UBQ10* and *PP2A* as internal standards. Asterisks and points indicate significant differences from the Col-0 and respective mock controls (* and • *p* < 0.05; ** and •• *p* < 0.01).

*ARR5*, a marker of CK action, was up-regulated in the mutants, although the fold induction was lower than in wild-type plants (Figure 4A). Three-h CK treatment of the mutants had no significant effect on the expression of *AHK2* and *AHK3*, which are predominant in green tissues [23]. In contrast, the *AHK4* expression was induced, both in the mutants and wild-type plants. Thus, the elevated steady-state levels of *ARR5* transcripts coincided in the mutants with increased basal expression levels of CK receptor gene *AHK4*. The baseline levels of transcript accumulation of type B response regulators *ARR1*, *ARR10* and *ARR12* and the transcriptional response to CK treatment were the same as in wild-type plants. 

To determine possible implication of other CK-responsive transcription factors, we examined the expression patterns of GATA factor gene *GNL* that was strongly up-regulated in Col-0, after CK treatment. Similar to wild type plants, the mutants exhibited elevated CK-induced transcript accumulation of this gene and that of *CRF6* (Figure 4B). *GNC*, *GLK1*, *GLK2*, and *HY5* did not respond to CK treatment, both in wild type and mutant plants. However, in the mutants, steady state levels of *GLK1* and *GLK2* were significantly lower and those of *HY5* and *GNL* higher than in Col-0. These results show that mutations of *PAP* genes may modulate transcript abundance of various CK-responsive transcription factor families, though regulatory circuit in response to hormone treatment remains generally unchanged.

**Figure 5:**
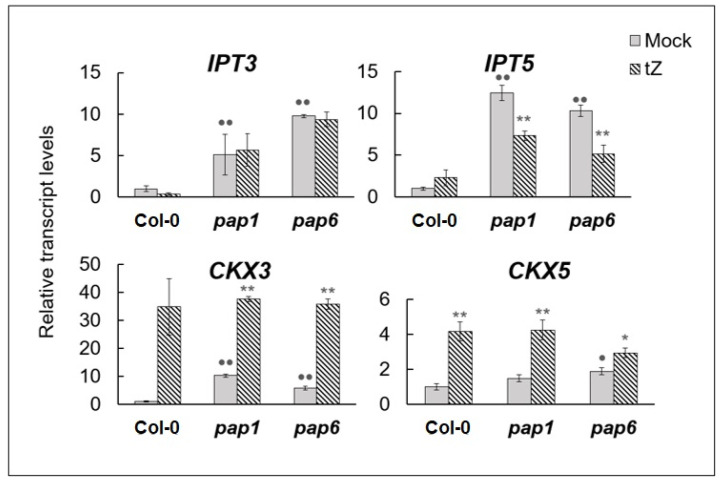
Relative expression values of *A. thaliana IPT* and *CKX* genes in untreated 4-week-old Col-0 plants and *pap* mutants, and under CK (*trans*-zeatin 5 μM, 3 h) treatment. *A. thaliana* Col-0 and mutant plants were grown on MS medium in Petri dishes for four weeks under a 16 h light/8 h dark photoperiod at 23 °C with 100 μE m^−2^ s^−1^. Total RNAs were isolated from rosette leaves and analyzed by relative quantitative RT-PCR using *UBQ10* and *PP2A* as internal standards. Asterisks and points indicate significant differences from the Col-0 and respective mock controls (* and • *p* < 0.05; ** and •• *p* < 0.01).

Elevated steady-state levels of ARR5 transcript, indicative of increased endogenous CK content [24] as well as transition from positive to negative type of CK dependent regulation of *PAP* genes allowed to suggest a change in the hormonal status of *pap* mutants. Therefore, the mutants were tested for potentially modified expression levels of selected cytokinin metabolic genes, as their transcript levels may correlate with hormone levels. All cytokinin biosynthesis genes isopentenyl-transferases (*IPT*) were reported to be weakly expressed under most conditions [1]. Moreover, expression of *IPT4, 6* and *8* was undetectable in vegetative tissues [25]. For this reason, two genes encoding plastid localized *IPT3* and *IPT5* enzymes were selected, which were shown to be involved in the synthesis of the bioactive tZ- and iP-type CKs. In both *pap* mutants, basal levels of these gene transcripts exhibited a strong increase as compared to wild type plants (Figure 5).

Modulation of CK levels can also be provided by CK degradation genes. Two of the seven cytokinin oxidase/dehydrogenase genes, *CKX3* and *CKX5* were selected, since their overexpression led to stronger phenotypic changes than that of other CKX genes [26,27]. Steady state levels of *CKX3* and *CKX5* transcripts were also elevated in the mutants though to a lesser extent, than those of *IPT* genes. Both *CKX3* and *CKX5* were strongly up-regulated by cytokinin in wild type (Figure 5), which is consistent with the microarray data [3], and much less in *pap* mutants. Overall, these findings allows us to expect that the altered response of *pap* mutants to exogenously applied cytokinin may be a consequence of the increased CK content.

### 3.3. PAP Mutants Have Increased Cytokinin Content 

Since some genes involved in hormone metabolism were differentially expressed in *pap1* and *pap6* the cytokinin levels were quantified in shoots of 5-week-old mutants and wild-type plant cultivated on MS medium. Both mutants showed a substantial increase in the total content of cytokinin metabolites (Table 1) with a clear predominance of precursors. The most noticeable changes (7-fold increase) were observed in the content of CK phosphates: *trans*-zeatin 9-riboside-5-monophosphate (ZRMP) and N6-(Δ2-isopentenyl) adenosine 5′-monophosphate (iPRMP). The concentration of active free bases *trans*-zeatin(tZ) and isopentenyladenine(iP) were probably below detectable limits though the levels of their corresponding ribosides appeared to be significantly higher in both mutants as compared to Col-0. However, Col-0 and the mutants contained approximately the same levels of dihydrozeatin (DZ) and CK storage forms: *trans*-zeatin riboside-O-glucoside (tZROG) and *cis*- zeatin riboside-O-glucoside (cZROG). CK-N-glucosides (tZ7G, tZ9G, iP7G and iP9G) the products of irreversible CK conjugation exhibited very high levels in all three samples and were roughly similar in Col-0 and *pap1* and a little less in *pap6*. Thus, elevated endogenous concentrations of CK metabolites might cause at least part of the differences in the response of chloroplast gene expression (Figure 5) to exogenously applied *trans*-zeatin.

### 3.4. CK-Regulated Expression of Chloroplast Encoded Genes and Nuclear Genes for Plastid Transcription Machinery Is Altered in the pap1 and pap6 Mutants

**Figure 6:**
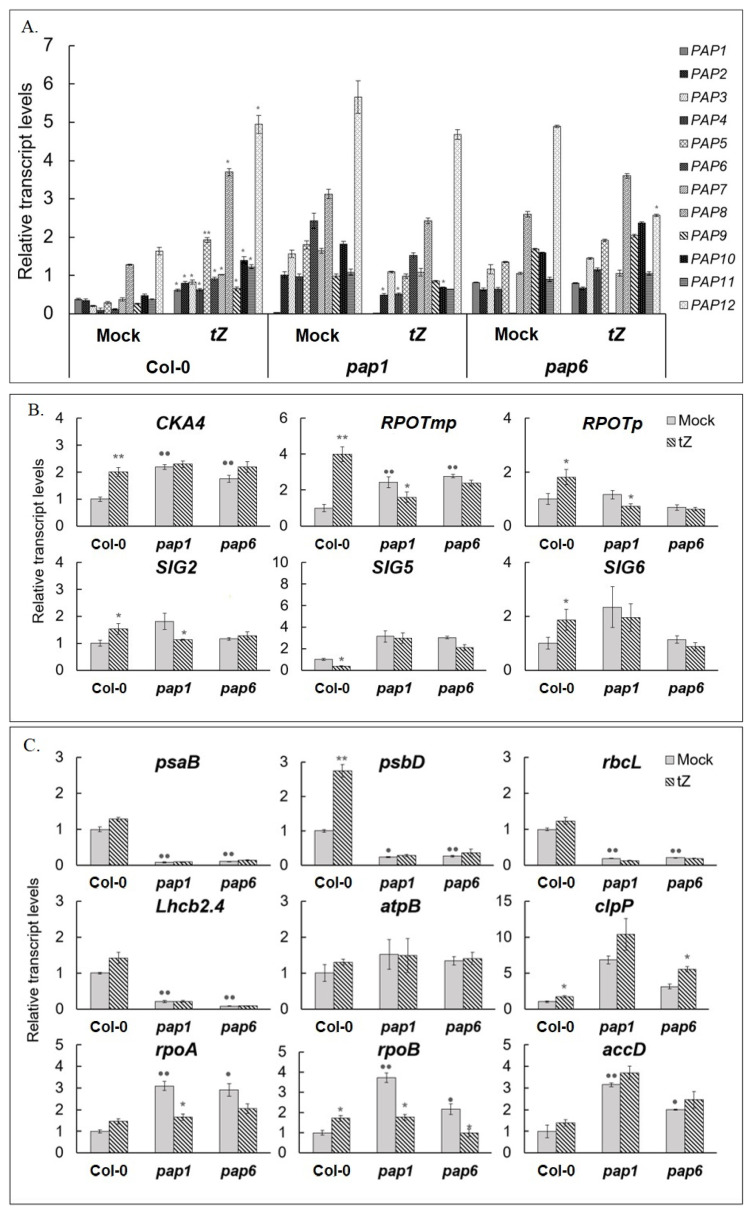
Relative expression values of *A. thaliana* nuclear genes for plastid transcription machinery (**A**,**B**) and chloroplast encoded genes (**C**) in untreated 4-week-old wild type plants and *pap* mutants, and under CK (*trans*-zeatin 5 μM, 3 h) treatment. *A. thaliana* Col-0 and mutant plants were grown on MS medium in Petri dishes for four weeks under a 16 h light/8 h dark photoperiod at 23 °C with 100 μE m^−2^ s^−1^. Total RNAs were isolated from rosette leaves and analyzed by relative quantitative RT-PCR using *UBQ10* and *PP2A* as internal standards. Asterisks and points indicate significant differences from the Col-0 and respective mock controls (* and • *p* < 0.05; ** and •• *p* < 0.01).

Before testing CK-dependent regulation of *PAP* genes in the mutants, the impact of disruption of a particular *PAP* gene on the expression of the remaining *PAP* genes was evaluated, as the altered genetic background can provoke an inadequate biological response to regulatory factor. According to our results, “knockout“ of *PAP1*, as well as of *PAP6*, contributed to the increase in the expression levels of at least some operative *PAP* genes (Figure 6A) which may indicate a modest compensatory up-regulation in response to constitutive deficiency. Similar data were previously presented for *pap10-1* mutant [28] in which steady state expression of PAPs tended to be up-regulated in addition to down-regulation in the pTAC10 overexpressing transgenic plants. Contrary to wild type plants, CK-treatment of the mutants did not significantly change transcript levels of the functioning *PAP* genes compared to their steady state levels or resulted in their slight decrease.

To further explore the effect of *pap* mutations on chloroplast transcription machinery we analyzed transcript levels of its counterparts. According to our prior studies *RPOTp*, *RPOTmp*, *SIG1*, *2, 6* and *CKA4* were induced and *SIG5* was repressed in Col-0 by CK treatment [12]. However, *RPOTp* and *RPOTmp*, were found to be either down-regulated or not affected by CK treatment in mutant lines (Figure 6B). Likewise, *SIG2, 5* and *6* and *CKA4* did not substantially change their transcript accumulation levels in the mutants or were even slightly inhibited. These findings suggest that disruption of *PAP* genes may alter the CK-dependent regulation of the genes for the plastid transcription machinery and as a consequence, change the response of plastid genes to treatment with exogenous hormone.

Chloroplast genes are categorized into three classes depending on selective transcription by PEP (class I), NEP (class III) or by both NEP and PEP (class II). To address specific changes in the CK-dependent accumulation of various plastid transcripts we compared the RNA profiles of genes belonging to all three classes. Transcript levels of class I (photosynthesis-related genes transcribed by PEP) are known to be considerably decreased in *pap* mutants [9]. In accordance with this premise, in both mutant lines tested, the expression levels of class I genes *psaB, psbD*, and *rbcL* were drastically reduced and were not significantly affected by CK, similarly to the expression pattern of nuclear-encoded *LHCB2.4* (light harvesting antenna protein of PSII) whose gene product is targeted to chloroplasts (Figure 6B). On the contrary, mRNA levels of class III genes which are enhanced in pap mutants [10,19,20,29] were either down-regulated (*rpoA, rpoB*) or did not respond to CK treatment (*accD*). The expression of class II gene *atpB* remained unaltered or slightly induced in the mutants and wild-type plants while that of *clpP* was up-regulated. These data are consistent with distinct transcriptional effects of cytokinin on particular genes, not related to a general impact on PEP- or NEP-dependent transcription.

### 3.5. Pap 1 Mutant Is Affected in the CK-Dependent Accumulation of Plastid Proteins

While the translation rates and accumulation of some organelle proteins could be associated with the levels of their corresponding mRNA, the steady state levels of many other proteins poorly correlate with accumulation of their transcripts [30,31]. To test whether CK dependent regulation of transcript levels in *pap1* mutant and Col-0 could fine tune the levels of their corresponding proteins, accumulation of selected plastid encoded proteins was analyzed.

**Figure 7:**
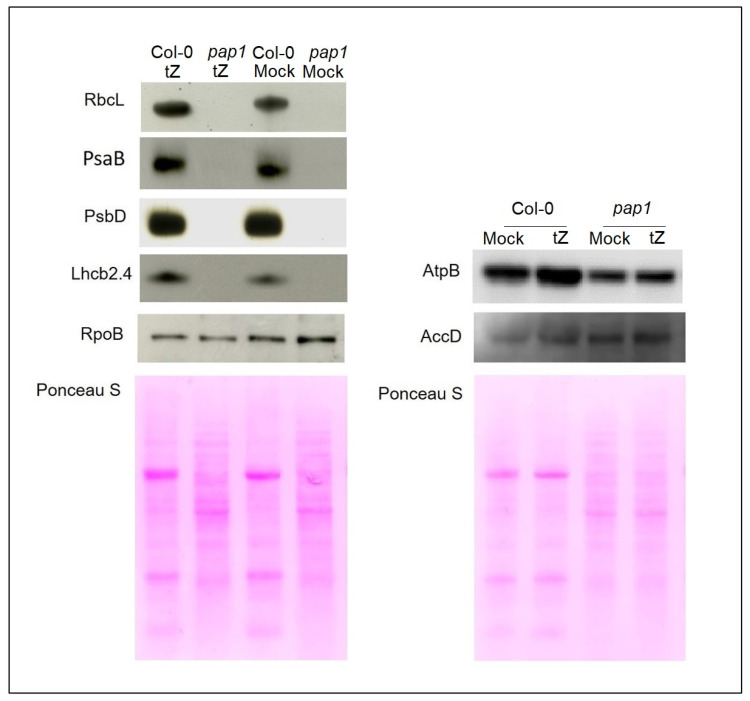
Immunoblot analysis of the photosynthetic proteins on the basis of equal total Ponceau S dye stained blot with proteins from leaves of wild type plants and *pap1* mutant grown on MS medium in Petri dishes for four weeks under a 16 h light/8 h dark photoperiod at 23 °C with 100 μE m^−2^ s^−1^. Proteins were visualized by immunoblotting using antibodies specific for RbcL, PsaB, PsbD, AtpB, RpoB, AccD and Lhcb2.4 proteins.

According to the results of immunoblot analysis, polypeptides of photosynthesis related genes RbcL (the large subunit of Rubisco), PsaB (PSI), PsbD (PSII) and LHCB2.4 (light harvesting antenna protein of PSII) were not detectable in pap1and remained below detectable levels even after extended cytokinin treatment (24 h, 5 × 10^−6^ M *trans*-zeatin) (Figure 7 and Supplementary Figure S1). We were also unable to trace reliably the accumulation of RPOTp proteins. They were reported to be present at very low levels in leaf chloroplasts, and were not detected in the proteomic analysis of whole chloroplast proteins or pTAC fractions [19].

On the contrary, AtpB (β subunit of ATP synthase complex) protein encoded by PEP and NEP dependent gene was easily discernible in *pap1* immunoblots, albeit at lower levels than in Col-0. Steady state protein levels of NEP dependent AccD (acetyl—CoA carboxylase subunit) did not differ in the mutant and wild-type plants and remained without notable effect when treated with CK.

A different pattern was obtained for RpoB (β-subunit PEP). In untreated mutant plants, the content of RpoB subunits was slightly increased compared to wild-type plants. However, it displayed a mild reduction after 24 h of CK treatment unlike Col-0, in which accumulation of RpoB slightly increased. Hence, disruption of *PAP1* promotes transition of CK-dependent accumulation of RpoB subunits from positive to negative.

Overall, our data show that CK-dependent shifts in chloroplast protein accumulation did not significantly differ from the changes of their RNA profiles.

## 4. Discussion

Elucidation of the mechanisms by which cytokinins regulate the expression of the genes for PEP-associated proteins may have a significant impact on the understanding of various aspects of transcriptional activity of chloroplasts during their development and functional maintenance. Given the effect of exogenous cytokinin treatment on the expression of chloroplast RNA polymerases *RPOTp* and *RPOTmp* and sigma factor genes, one would expect that *PAP* genes are also regulated by CK. Indeed, according to our results *PAP* genes were significantly up-regulated by CK at different time points of plant development and with a maximum impact at the late stages of ontogenesis. Hence, the effectiveness of the applied cytokinin depends on its endogenous levels, having more profound effect in the case of low CK levels, which occur in senescing leaves [32].

The transcriptional response to CK is mediated via a canonical cytokinin signaling pathway including cytokinin receptors AHK2, AHK3 and AHK4, phosphor-transfer proteins AHP 2, 3, and 5 and type-B ARR proteins 1, 10 and 12. According to our previous results, the level of transcript accumulation of *PAP5* gene did not change in the *arr1,10,12* or *ahk2,3* background when the mutants were grown on a CK containing medium (10^−6^ M *trans*-zeatin) in the dark and during the first hours of de-etiolation [21]. Thus, growth specific conditions and developmental stage did not affect the involvement of specific CK receptors or type-B response regulators in CK-induced regulation of *PAP* genes. 

The *PAP* genes may be affected in parallel by CRF proteins, which receive input from cytokinin receptors or from non-cytokinin-dependent signaling sources, or by other signaling pathways (e.g., by ethylene) [4]. However, direct interaction between *PAP* genes and diverse CK regulated transcription factors remains to be shown. With the exception of *PAP8* and *PAP11*, no such interactions were found so far in vivo between type-B ARR1, 10 and 12 and PAP genes [33]. The technique used by Xie et a1. (2018) combined Chip-seq analysis and the employment of plants, which contained epitope-tagged type*-B* ARRs permitting to identify their genome-wide binding locations [33]. Their results partly confirm the absence of binding motifs for ARR1, 10 and 12 obtained by *in silico* analysis of *PAP cis*-acting elements (Table S2). Nevertheless, PAPs may be the direct targets for other type-B ARRs, for example, ARR11 or ARR14 which are apparently able to bind the promoters of most PAP genes. 

*Trans*-factors (TF) binding does not necessary coincide with changes in gene transcription [33,34]. Thus, the transcriptional responses driven by B-ARRs may be indirect since several CK-related TFs that can potentially interact with the promoters of *PAP* genes (i.e., ARR14, GLK1, HY5 and GNC) can be found in the type-B ARR target gene list [33]. In this regard, it is of interest that up-regulation of *PAP*s reached maximum after 3 h of CK application and was maintained at elevated levels for at least 24 h, indicative of indirect control. In fact, such regulation may represent a collection of diverse regulatory pathways formed by related suits of genes and none of them applies to all cases.

For example, coordinated expression of *PAP* genes in the early stages of chloroplast biogenesis could be governed by HY5, as proposed by Liebers et al. [15]. HY5, a basic leucine zipper transcription factor, detectably affects the expression of over 1100 genes, including the target genes related to cytokinin pathway [35]. The expression profile of *HY5* parallels the transcript accumulation of *PAP* genes during skoto- to photomorhogenesis transition. [15]. Moreover, inactivation of *HY5* caused almost complete absence of CK-dependent elevation of *PAP* gene expression during de-etiolation of dark-grown *hy5* seedlings [36], but under standard illumination the blocking effect of the *hy5* mutation on the CK-induced increase in the expression of *PAP* genes was absent. It should be noted that in our model, *HY5* did not directly respond to CK treatment suggesting participation of additional master regulators. Overall, these data indicate that CK can govern the transcriptional regulation of *PAP* genes through CK related signaling network in a context dependent manner.

Despite chloroplast deficiency, the CK dependent regulatory circuit in *pap* mutants remains unperturbed though disruption of *PAP* genes may modulate steady state transcript levels of various CK-responsive transcription factor families. Thus, the expression of *CRF6* that acts in parallel with GATA transcription factor gene *GNL* was strongly and rapidly up-regulated by CK both in *pap* mutants and Col-0. The induction of *GNC* was less manifested in accordance with the data presented for this *trans*-factor by Chiang et al. [7]. These observations suggest that CK-mediated signaling network is still functional in both *pap* mutants although they display all the molecular and structural features referred to as the “PAP syndrome”. In sum, the result is consistent with the earlier report that photomorphogenetic program in *pap7-1* mutant of *Arabidopsis thaliana* appears to be operational as long as an external carbon source is available [10].

An arrest of chloroplast biogenesis in *pap* mutants provokes enhanced accumulation of NEP-dependent gene transcripts and reduced accumulation of transcripts of PEP-transcribed genes, similar to *rpo* deletion mutants [19,29]. In our experiments, PEP-dependent photosynthesis genes exhibited a dramatic decrease at the level of transcript accumulation in *pap* mutants. They were either inhibited by CK treatment or did not respond to it, as opposed to the wild type. This suggests that the CK-dependent regulation of class I genes depends on the normal formation of the PEP complex, which determines the development and functioning of chloroplasts. Herewith, an altered biological response could not be rescued by compensatory enhancement of NEP activity which was shown to be able to transcribe even photosynthesis genes from remote promoters in albino mutants [8,9]. Moreover, we found that the expression of NEP-dependent chloroplast genes *accD, rpoA* and *rpoB* and nuclear encoded components of the plastid transcription machinery were also down-regulated by cytokinin or were not significantly altered by CK treatment (Figure 6). In addition, CK-dependent shifts of RNA profiles were accompanied by mild reduction in protein accumulation for class III house-keeping genes (Figure 7). An altered biological response to CK treatment in *pap* mutants was also demonstrated in dark detached leaf senescence assay (Figure 3). These results imply that depending on the functional state of the chloroplast, CK response can be converted from positive to negative or vice-versa.

Opposite reaction to cytokinin treatment in the mutants with the disrupted *PAP* genes may be relevant with the hormone overproduction as a consequence of the strongly up-regulated expression of the genes for CK synthesis (Figure 5, Table 1). Apparently, such a biological response is not restricted to impairment of *PAP* genes per se but could be associated with the arrest of chloroplast biogenesis by a variety of damaging mechanisms. Similar effect was revealed in white leaves of barley mutant *albostrians* where chloroplast development was completely blocked due to a nuclear mutation causing a lack of plastid ribosomes [37]. White leaves contained higher levels of cytokinins then green leaves and were less sensitive to CK promoted stomatal opening and protein retention in dark-detached leaf segments. Despite the fact that ^35^S-methionine incorporation into leaf proteins was higher in white leaves, CK-mediated effect on methionine incorporation and hence protein synthesis was much more pronounced in green leaves.

Therefore, unimpaired chloroplast development is required for normal responses of plants to cytokinins. However, exact reasons for higher CK levels in white plants and the adaptive outcome, if any, are still obscure. Elevated CK content may be the result of a certain compensatory mechanism allowing plants to promote morphogenesis in the absence of normal photosynthesis. The effect of cytokinins on the de-etiolation response in plastids of dark-grown *Arabidopsis thaliana* plants was first demonstrated by Chory et al. [38]. The seedlings in presence of CKs developed large lens-shaped plastids that lacked a prolamellar body and contained some bi-thylakoid membranes. They formed normal rosettes, similar to *pap* mutants grown on a sugar-containing medium but were unable to produce seeds. 

Reprogramming of CK-metabolic genes and, consequently, a change in CK-status may result from retrograde signaling from the dysfunctional chloroplast through a multiplicity of pathways involving transcriptional repressors, chromatin remodeling and alternative splicing [6]. For example, plastid signals were found to repress the light-regulated genes involved in the jasmonic acid biosynthesis and signaling, through AtMYC2 (MYC-related transcriptional activator) which is known to act as a positive regulator of jasmonic acid signaling and a negative regulator of blue light signaling [39]. Whether similar mechanism could account for increased cytokinin content in *pap* mutants remains to be found. It should be noted, however, that in *pap7-1* mutants the structural and functional defect in the PEP-PAP complex did not affect the accumulation of other nuclear-encoded components of the plastid transcription machinery which allowed the authors to suppose that these genes were not under retrograde control [10]. Nonetheless, both retrograde and anterograde signaling involve components from the pathways crossing each other at different junctions [39] and their interplay may define the appropriate response of plants to hormonal treatment. This is especially true for *PAP* genes with their versatile structure of promoter regions imposed on different functional and structural features.

## 5. Conclusions

Overall, our findings provide evidence that *PAP* genes are involved in CK-mediated regulation of the chloroplast transcription machinery via canonical CK signaling pathway. However, this regulation may be indirect. Changes in the mode of the response to cytokinin treatment are affected by the functional state of plastids. In *pap* mutants with the arrested albino plastids and the increased levels of endogenous CKs, the effect of CK treatment can change from positive to negative and contribute to the reduction in accumulation of transcripts for chloroplast genes and genes for plastid transcription machinery.

## Figures and Tables

**Table 1:** The impact of *pap* mutations on CK content (pmol/g fresh weight) of *Arabidopsis thaliana* 5-week-old rosette leaves.

Line	DZ	tZR	iPR	cZR	CK Ribosides	ZRMPt	iPRMP	cZRMP	CK Phosphates
Col-0	1.40 ± 0.60	20.37 ± 4.74	1.04 ± 0.60	1.23 ± 0.62	22.64 ± 5.76	9.69 ± 4.94	12.38 ± 3.44	5.63 ± 1.96	27.70 ± 9.07
*pap1*	0.43 ± 0.43	31.28 ± 3.42 *	4.36 ± 1.75 *	2.87 ± 2.13	38.50 ± 4.77 *	71.57 ± 9.95 **	97.40 ± 26.79 **	5.48 ± 2.81	174.44 ± 32.57 **
*pap6*	2.2 ± 0.38	23.43 ± 4.46	9.59 ± 2.95 **	0.62 ± 0.42	33.64 ± 6.57	71.53 ± 17.82 **	89.84 ± 32.28 **	6.98 ± 3.48	168.35 ± 51.31 **
Line	tZROG	cZROG	CK-O glucosides	tZ7G	tZ9G	iP7G	iP9G	cZ7G	CK N glucosides
Col-0	4.20 ± 1.70	10.16 ± 2.11	14.35 ± 3.56	143.11 ± 31.95	33.39 ± 13.33	372.59 ± 134.58	12.23 ± 5.54	82.66 ± 25.47	643.99 ± 191.84
*pap1*	6.71 ± 0.84	10.12 ± 4.49	16.83 ± 4.48	114.11 ± 20.62	33.22 ± 4.39	418.30 ± 36.34	12.89 ± 5.30	75.44 ± 38.30	653.97 ± 56.98
*pap6*	5.90 ± 1.22	7.94 ± 1.45	13.84 ± 2.60	80.08 ± 11.72 *	19.31 ± 8.17	359.95.95 ± 53.39	10.93 ± 3.09	30.66 ± 13.89 *	500.93 ± 69.43

Designations: CK bases: DZ—dihydrozeatin; CK ribosides: tZR—*trans*-zeatin riboside; iPR—*N*6-(2-isopentenyl) adenine riboside; cZR—*cis*-zeatin riboside. CK phosphates: ZRMP—trans-zeatin riboside monophosphate; iPRMP—isopentenyladenosine riboside monophosphate; cZR—*cis*-zeatin riboside monophosphate; CK O-glucosides: tZROG—*trans*-zeatin/riboside O-glucoside; cZROG—*cis*-zeatin/riboside O-glucoside. CK N- glucosides: tZ7G—*trans-*zeatin 7-glucoside; tZ9G—*trans*-zeatin 9-glucoside; iP7G and iP9G—isopentenyladenine N7- and N9 glucosides, cZ7G—*cis*-zeatin N7-glucoside dihydrozeatin N7- and N9-glucosides. Asterisks indicate significant differences from the Col-0 (* *p* < 0.05; ** *p* < 0.01).

## Supplementary Materials

The following are available online at https://www.mdpi.com/2218-273X/10/12/1658/s1, Figure S1: Immunoblot analysis of the photosynthetic proteins, Table S1: Primers sequences used in this study for qRT-PCR; Table S2: List of CK-related transcription factors identified in promoters of Pap genes using AthaMap server.

## References

[1] Brenner W.G., Ramireddy E., Heyl A., Schmülling T. (2012). Gene regulation by cytokinin in Arabidopsis. Front. Plant Sci..

[2] Bhargava A., Clabaugh I., To J.P., Maxwell B.B., Chiang Y.H., Schaller G.E., Kieber J.J. (2013). Identification of cytokinin-responsive genes using microarray meta-analysis and RNA-Seq in Arabidopsis. Plant Physiol..

[3] Brenner W.G., Schmülling T. (2015). Summarizing and exploring data of a decade of cytokinin-related transcriptomics. Front. Plant Sci..

[4] Rashotte A.M., Mason M.G., Hutchison C.E., Ferreira F.J., Schaller G.E., Kieber J.J. (2006). A subset of Arabidopsis AP2 transcription factors mediates cytokinin responses in concert with a two-component pathway. Proc. Natl. Acad. Sci. USA.

[5] Cortleven A., Schmülling T. (2015). Regulation of chloroplast development and function by cytokinin. J. Exp. Bot..

[6] Zubo Y.O., Blakley I.C., Franco-Zorrilla J.M., Yamburenko M.V., Solano R., Kieber J.J., Schaller G.E. (2018). Coordination of chloroplast development through the action of the GNC and GLK transcription factor families. Plant Physiol..

[7] Chiang Y.H., Zubo Y.O., Tapken W., Kim H.J., Lavanway A.M., Howard L., Schaller G.E. (2012). Functional characterization of the GATA transcription factors GNC and CGA1 reveals their key role in chloroplast development, growth, and division in Arabidopsis. Plant Physiol..

[8] Zhelyazkova P., Sharma C.M., Förstner K.U., Liere K., Vogel J., Börner T. (2012). The primary transcriptome of barley chloroplasts: Numerous noncoding RNAs and the dominating role of the plastid-encoded RNA polymerase. Plant Cell.

[9] Pfannschmidt T., Blanvillain R., Merendino L., Courtois F., Chevalier F., Liebers M., Lerbs-Mache S. (2015). Plastid RNA polymerases: Orchestration of enzymes with different evolutionary origins controls chloroplast biogenesis during the plant life cycle. J. Exp. Bot..

[10] Grübler B., Merendino L., Twardziok S.O., Mininno M., Allorent G., Chevalier F., Ravanel S. (2017). Light and plastid signals regulate different sets of genes in the albino mutant pap7-1. Plant Physiol..

[11] Yagi Y., Shiina T. (2014). Recent advances in the study of chloroplast gene expression and its evolution. Front. Plant Sci..

[12] Danilova M.N., Kudryakova N.V., Doroshenko A.S., Zabrodin D.A., Rakhmankulova Z.F., Oelmüller R., Kusnetsov V.V. (2017). Opposite roles of the Arabidopsis cytokinin receptors AHK2 and AHK3 in the expression of plastid genes and genes for the plastid transcriptional machinery during senescence. Plant Mol. Biol..

[13] Bastakis E., Hedtke B., Klermund C., Grimm B., Schwechheimer C. (2018). LLM-domain B-GATA transcription factors play multifaceted roles in controlling greening in Arabidopsis. Plant Cell.

[14] Borsellino L. (2012). Influence of Light and Cytokinin on Organellar Phage-Type RNA Polymerase Transcript Levels and Transcription of Organellar Genes in *Arabidopsis thaliana*. Ph.D. Thesis.

[15] Liebers M., Chevalier F., Blanvillain R., Pfannschmidt T. (2018). PAP genes are tissue-and cell-specific markers of chloroplast development. Planta.

[16] Dobrev P.I., Kamınek M. (2002). Fast and efficient separation of cytokinins from auxin and abscisic acid and their purification using mixed-mode solid-phase extraction. J. Chromatogr. A.

[17] Dobrev P.I., Vankova R. (2012). Quantification of abscisic acid, cytokinin, and auxin content in salt-stressed plant tissues. Methods Mol. Biol..

[18] Steffens N.O., Galuschka C., Schindler M., Bülow L., Hehl R. (2005). AthaMap web tools for database-assisted identification of combinatorial cis-regulatory elements and the display of highly conserved transcription factor binding sites in Arabidopsis thaliana. Nucleic Acids Res..

[19] Yagi Y., Ishizaki Y., Nakahira Y., Tozawa Y., Shiina T. (2012). Eukaryotic-type plastid nucleoid protein pTAC3 is essential for transcription by the bacterial-type plastid RNA polymerase. Proc. Natl. Acad. Sci. USA.

[20] Gilkerson J., Perez-Ruiz J.M., Chory J., Callis J. (2012). The plastid-localized pfkB-type carbohydrate kinases fructokinase-like 1 and 2 are essential for growth and development of Arabidopsis thaliana. BMC Plant Biol..

[21] Danilova M.N., Kudryakova N.V., Andreeva A.A., Doroshenko A.S., Pojidaeva E.S., Kusnetsov V.V. (2018). Differential impact of heat stress on the expression of chloroplast-encoded genes. Plant Physiol. Biochem..

[22] He L., Zhang S., Qiu Z., Zhao J., Nie W., Lin H., Zhu L. (2018). FRUCTOKINASE-LIKE PROTEIN 1 interacts with TRXz to regulate chloroplast development in rice. J. Integr. Plant Biol..

[23] Werner T., Schmuülling T. (2009). Cytokinin action in plant development. Curr. Opin. Plant Biol..

[24] Aloni R., Langhans M., Aloni E., Dreieicher E., Ullrich C.I. (2005). Root-synthesized cytokinin in Arabidopsis is distributed in the shoot by the transpiration stream. J. Exp. Bot..

[25] Miyawaki K., Tarkowski P., Matsumoto-Kitano M., Kato T., Sato S., Tarkowska D., Kakimoto T. (2006). Roles of Arabidopsis ATP/ADP isopentenyltransferases and tRNA isopentenyltransferases in cytokinin biosynthesis. Proc. Natl. Acad. Sci. USA.

[26] Werner T., Motyka V., Laucou V., Smets R., Van Onckelen H., Schmülling T. (2003). Cytokinin-deficient transgenic Arabidopsis plants show multiple developmental alterations indicating opposite functions of cytokinins in the regulation of shoot and root meristem activity. Plant Cell.

[27] Ha S., Vankova R., Yamaguchi-Shinozaki K., Shinozaki K., Tran L.S.P. (2012). Cytokinins: Metabolism and function in plant adaptation to environmental stresses. Trends Plant Sci..

[28] Chang S.H., Lee S., Um T.Y., Kim J.K., Do Choi Y., Jang G. (2017). pTAC10, a key subunit of plastid-encoded RNA polymerase, promotes chloroplast development. Plant Physiol..

[29] Arsova B., Hoja U., Wimmelbacher M., Greiner E., Üstün Ş., Melzer M., Börnke F. (2010). Plastidial thioredoxin z interacts with two fructokinase-like proteins in a thiol-dependent manner: Evidence for an essential role in chloroplast development in Arabidopsis and Nicotiana benthamiana. Plant Cell.

[30] Kusnetsov V.V., Oelmüller R., Sarwat M.I., Porfirova S.A., Cherepneva G.N., Herrmann R.G., Kulaeva O.N. (1994). Cytokinins, abscisic acid and light affect accumulation of chloroplast proteins in Lupinus luteus cotyledons without notable effect on steady-state mRNA levels. Planta.

[31] Shevtsov S., Nevo-Dinur K., Faigon L., Sultan L.D., Zmudjak M., Markovits M., Ostersetzer-Biran O. (2018). Control of organelle gene expression by the mitochondrial transcription termination factor mTERF22 in Arabidopsis thaliana plants. PLoS ONE.

[32] Yamburenko M.V., Zubo Y.O., Vanková R., Kusnetsov V.V., Kulaeva O.N., Börner Th. (2013). Abscisic acid represses the transcription of chloroplast genes. J. Exp. Bot..

[33] Xie M., Chen H., Huang L., O’Neil R.C., Shokhirev M.N., Ecker J.R. (2018). A B-ARR-mediated cytokinin transcriptional network directs hormone cross-regulation and shoot development. Nat. Commun..

[34] MacQuarrie K.L., Fong A.P., Morse R.H., Tapscott S.J. (2011). Genome-wide transcription factor binding: Beyond direct target regulation. Trends Genet..

[35] Zhang H., He H., Wang X., Wang X., Yang X., Li L., Deng X.W. (2011). Genome-wide mapping of the HY5-mediated genenetworks in Arabidopsis that involve both transcriptional and post-transcriptional regulation. Plant J..

[36] Doroshenko A.S., Danilova M.N., Andreeva A.A., Kudryakova N.V., Kuznetsov V.V., Kusnetsov V.V. (2020). The Transcription Factor HY5 Is Involved in the Cytokinin-Dependent Regulation of the Expression of Genes Encoding Proteins Associated with Bacterial Plastid RNA-Polymerase during De-Etiolation of Arabidopsis thaliana. Dokl. Biochem. Biophys..

[37] Kulaeva O.N., Burkhanova E.A., Karavaiko N.N., Selivankina S.Y., Porfirova S.A., Maslova G.G., Börner T. (2002). Chloroplasts affect the leaf response to cytokinin. J. Plant. Physiol..

[38] Chory J., Reinecke D., Sim S., Washburn T., Brenner M. (1994). A role for cytokinins in de-etiolation in Arabidopsis (det mutants have an altered response to cytokinins). Plant. Physiol..

[39] Burman N., Khurana J.P. (2013). Photoregulation of Chloroplast Development: Retrograde Signaling. Plastid Development in Leaves during Growth and Senescence.

