# Supplementary material for: Cytokinin-Regulated Expression of Arabidopsis thaliana PAP Genes and Its Implication for the Expression of Chloroplast-Encoded Genes
Source: Biomolecules. 2020 Dec 11;10(12):1658. doi: 10.3390/biom10121658 (PMC7764210; doi:10.3390/biom10121658)
Supplement: Supplementary file 1 [file biomolecules-10-01658-s001.pdf]

Cytokinin-regulated Expression of *Arabidopsis thaliana PAP* Genes and its Implication for the Expression of Chloroplast-encoded Genes

Aleksandra A. Andreeva [1], Radomira Vankova [2], Ivan A. Bychkov [1], Natalia V. Kudryakova [1,*], Maria N. Danilova [1], Jozef Lacek [2], Elena S. Pojidaeva[1], Victor V. Kusnetsov [1]

[1] Timiryazev Institute of Plant Physiology, Russian Academy of Sciences, Botanicheskaya 35, Moscow, 127276, Russia

[2] Institute of Experimental Botany, Academy of Sciences CR, Rozvojova 263, 165 02 Prague 6, Czech Republic

Correspondence: nvkudryakova@mail.ru; Tel.: + 7 (499) 678-54-00

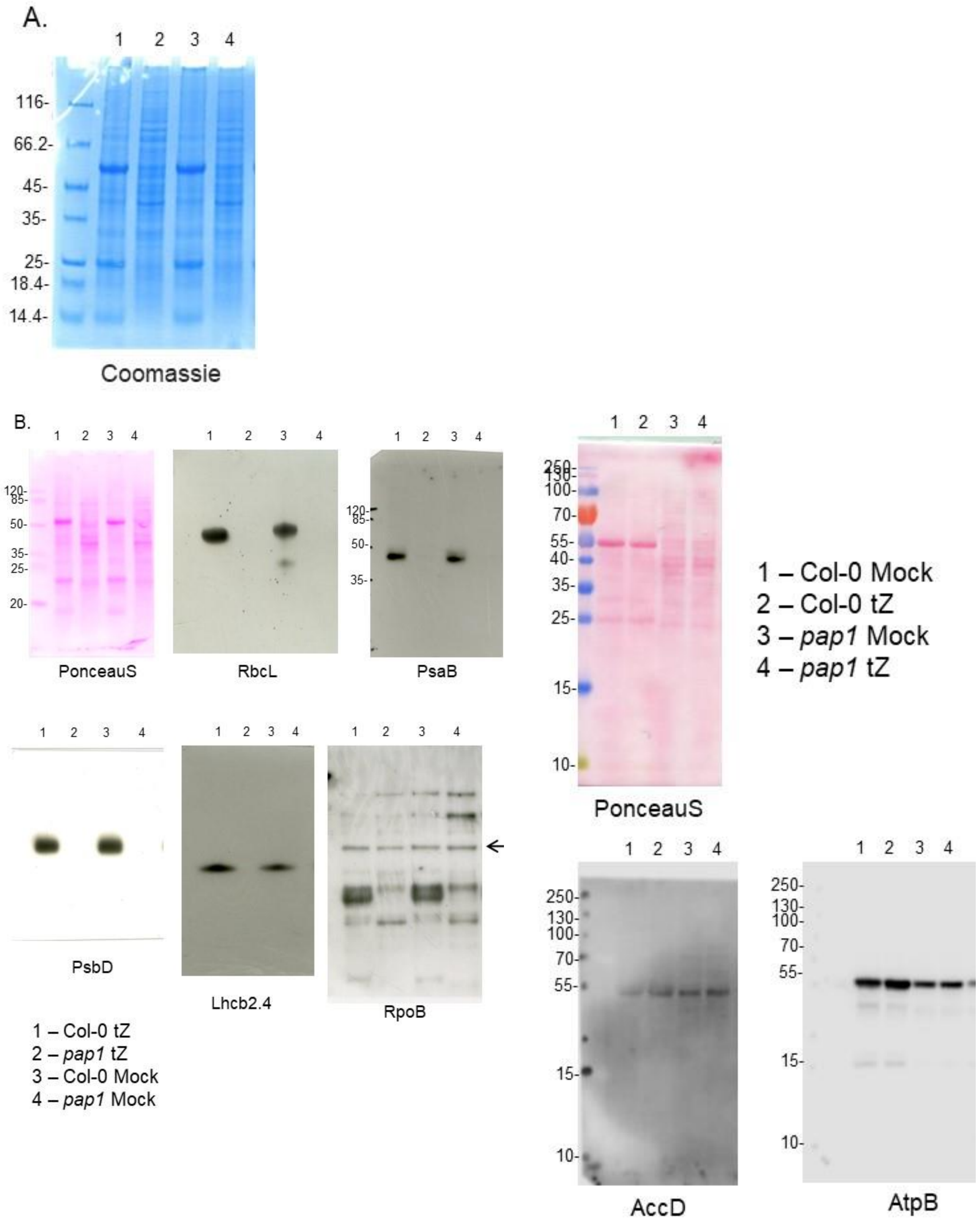

Figure S1. Immunoblot analysis of the photosynthetic proteins on the basis of equal total Ponceau S dye stained blot with proteins from leaves of wild type plants and *pap1* mutant grown on MS medium in Petri dishes for four weeks under a 16 h light/8 h dark photoperiod at 23 °C with 100 μE m^{-2} s^{-1}. Proteins were visualized by immunoblotting using antibodies specific for RbcL, PsaB, PsbD, AtpB, RpoB, AccD and Lhcb2.4 proteins.

Table S1. Primers sequences used in this study for qRT-PCR

Gene (Locus)	Forward/Reverse	Sequence (5′–3′)
PAP1 (AT3G04260)	Forward	TGAGAGTGGTGGAGAGATTACGGAA
	Reverse	CCATCTTGCTTGCTGCTTTCG
PAP2 (AT1G74850)	Forward	CGGGGACAGTTGGAGAAAAGC
	Reverse	GACCTCCATTCCATCCCGTGA
PAP3 (AT3G48500)	Forward	GGAAAGGCAATGTGTGGTATGA
	Reverse	AAGAGGTTGCTGTTCCGAGTAA
PAP4 (AT5G23310)	Forward	ATCAAGGCTACATACAACAACGGGA
	Reverse	AACCAAAATCCTTATCAATCTGCTCAA
PAP5 (AT2G34640)	Forward	ATGCGGTTATGGATTTCAGAG
	Reverse	TTCTCTTTTGTCAGTAGTGCTTCAT
PAP6 (AT3G54090)	Forward	GGCGGAGACTGTGAAAGAACCA
	Reverse	GTATTGCCGTAAAAAGAGTTGATTGC
PAP7 (AT4G20130)	Forward	TATGGGCGGTTAGTATGGCACAG
	Reverse	CAGCATTTGACATAACCTCCAGCA
PAP8 (AT1G21600)	Forward	CACTGAACCACCTATTGATGCCC
	Reverse	GAACTCTGGCTCTGGTGATTGACTT
PAP9 (AT5G51100)	Forward	GCAGTAAATCCGCTCGTATGGG
	Reverse	CATCTTCTGTCTCTGTTCCTTCTTGTTCT
PAP10 (AT3G06730)	Forward	AGGTGCCGTTGATTGTTGAT
	Reverse	GGACTGATGAAGAATAATGTAGGTAAC
PAP11 (AT1G63680)	Forward	CGTTGTGCTGTTGCTATGGGTG
	Reverse	TGCTTCCCGACATTCCTCTCTG
PAP12 (AT5G24314)	Forward	AGAGAGATGATAATGGACGCCG
	Reverse	TGTCATTACCTTCCCAACTTCTCTT
ARR5 (AT3G48100)	Forward	CTA CTC GCA GCT AAA ACG C
	Reverse	GCC GAA AGA ATC AGG ACA
RpoTp (AT2G24120)	Forward	TTG CTG CTG CTT GCT ATT CTG C
	Reverse	GCA CAA TCA CCA AGC CAA CT
RpoTmp (AT5G15700)	Forward	CGT TTC CTC ATT TAG ACT TTC CTC C
	Reverse	CCT TCT CTC TGT CTG CGT CTC TGT
AHK2 (AT5G35750)	Forward	GGG GAT ATA AGT GAG CAG CAT ATA A
	Reverse	TGT CCA GGG AAT GGG AAG TT
AHK3 (AT1G27320)	Forward	GCA TCG GAG CTT TGA ACC AT
	Reverse	GGA TAT GGA TGG TCC GAC TTG
AHK4 (AT2G01830)	Forward	ATT CTA GCG ATG ACT GCG GA
	Reverse	CGA CGA AGG TGA GAT AGG ATT
ARR1 (AT3G16857)	Forward	ACA ACG GAA TGC TGA TGC CTC T
	Reverse	CAC TCT GCT GCT CTC GGG GAT
ARR10 (AT4G31920)	Forward	AGT GGT TTG ATG GCT TCT GAT GC

	Reverse	AAA TCC CAA TCA CCT TCC GAG A
ARR12 (AT2G25180)	Forward	GTT TCC ATC ACC GCC CAA TC
	Reverse	CCG AAG GAG TAT TGA GTC TGC CA
SIG2 (AT1G08540)	Forward	TCT TCT TCG TCT TCA TCA TCC GC
	Reverse	CTG CTG CTG CTA CAA CTA CTG CTT
SIG5 (AT5G24120)	Forward	AGA TGT TGA TGG TGT TGG AGC
	Reverse	GAC TCT CTT TCG GCT TCA ATG
SIG6 (AT2G36990)	Forward	TCG CCT ATT GTT GGT TCG C
	Reverse	GGG CTG ATA ATG ATG ATG CG
psbA (ArthCp002)	Forward	CGG TGC CAT TAT TCC TAC TTC T
	Reverse	CTT GCC CGA ATC TGT AAC CT
psbD (ArthCp017)	Forward	GGA TGA CTG GTT ACG GAG GG
	Reverse	GGT TGT ACC TGT GAA CCA ACC
rbcL (ArthCp030)	Forward	CGG GTA CAT GCG AAG AAA TGA
	Reverse	TCT CGG TCA AAG CAG GCA TA
LHCB2.4 (AT3G27690)	Forward	CCA ACG ATC TCC TCC GCA AA
	Reverse	AGA CTT GAC GGT ACG ACG CA
atpB (AtCg00480)	Forward	CTA TGA GTG CGA CAG AGG GT
	Reverse	GAT AGG AGA TGT TGT GCG AG
clpP (ArthCp048)	Forward	CAT TCC AGA TAT TAC CCA TCC A
	Reverse	GCC AAG AGG TTG ATA CCG AA
rpoA (ArthCp055)	Forward	CGC CAA GTA AAG CTC TTC GC
	Reverse	AAG GCC AAG CCG ACA CAA TA
rpoB (ArthCp014)	Forward	ATG AGC AAC ACC AAA CCC CT
	Reverse	GGG TAG GCG AAA TGG AGG TT
accD (ArthCp031)	Forward	GCT ACC AAT CAA TGT TTA CCT C
	Reverse	GAT TGA TAA TCA CAT AAA ACC G
GNL (AT4G26150)	Forward	CCA TAT CTC CCA ACC TCT CG
	Reverse	TGG GCA CCA TTT GAT CAC T
GNC (AT5G56860)	Forward	GCG TGA TTA GGG TTT GTT CG
	Reverse	CTT TGC CGT ATA CCA CAT GC
GLK1 (At2g20570)	Forward	CCG GTA GAC TTA CAT CCG TCA
	Reverse	CAT GGC CTC GTC AAT ACA TCT
GLK2 (AT5G44190)	Forward	AAC GTT GCT AGC CAT CTT CAG
	Reverse	CTC CTA CTC CGG GCA CTG
CRF6 (AT3G61630)	Forward	GACGAACGAGACGAGTGAAGT
	Reverse	ACGAGTCAGTAGCGAAAGGAT
HY5 (AT5G11260)	Forward	CCA TCA AGC AGC GAG AGG TCA T
	Reverse	CGC CGA TCC AGA TTC TCT ACC G
CKX3 (AT5G56970)	Forward	TCT CAA TAC ACA GTC AAC GAG GA
	Reverse	TCG TAC ATA AAC CCT CTT ACA TGG
CKX5 (AT1G75450)	Forward	CCA TGG TCC TCA AAT TAG TAA CG

	Reverse	TCT GAG CAT CTC ATC ACC TCT C
IPT3 (AT3G63110)	Forward	AGT GGA ATG GTT GAG GAA GTC
	Reverse	CAA GAA CTG CTC GTT CCT GA
IPT5 (AT5G19040)	Forward	AGT TAC AGC GAT GAC CAC CA
	Reverse	GGC AGA GAT CTC CGG TAG G
CKA4 (AT2G23070)	Forward	TCT TTT ATG GCC ATG ACA ACT
	Reverse	CTG TTG GCC TTT CTT GGT

Table S2. List of CK-related transcription factors identified in promoters of *Pap* genes using AthaMap server

Gene	Factor	Family	Core binding motif
Pap1	ARR11	ARR-B	AGAT
	ARR14	ARR-B	AGAT
	GNC	GATA	GATC
	GLK1	GARP	CCAATC
	HY5	bZIP	ACGT
PAP2	ARR10	ARR-B	AGAT
	ARR11	ARR-B	AGAT
	ARR14	ARR-B	AGAT
	GLK1	GARP	CCAATC
PAP3	ARR10	ARR-B	AGAT
	ARR11	ARR-B	AGAT
	ARR14	ARR-B	AGAT
	GLK 1	GARP	CCAATC
PAP4	ARR11	ARR-B	AGAT
	ARR14	ARR-B	AGAT
	GLK1	GARP	CCAATC
PAP5	ARR11	ARR-B	AGAT
	ARR14	ARR-B	AGAT
	GLK1	GARP	CCAATC
PAP6	ARR11	ARR-B	AGAT
	ARR14	ARR-B	AGAT
	GLK1	GARP	CCAATC
PAP7	ARR11	ARR-B	AGAT
	ARR14	ARR-B	AGAT
	GLK1	GARP	CCAATC
PAP8	ARR10	ARR-B	AGAT
	ARR11	ARR-B	AGAT
	ARR14	ARR-B	AGAT
	GLK1	GARP	CCAATC
PAP9	ARR10	ARR-B	AGAT
	ARR11	ARR-B	AGAT
	GLK1	GARP	CCAATC
PAP10	ARR11	ARR-B	AGAT
	ARR14	ARR-B	AGAT
	GLK1	GARP	CCAATC
PAP11	ARR1	ARR-B	AGAT
	ARR10	ARR-B	AGAT
	ARR11	ARR-B	AGAT
	GLK1	GARP	CCAATC
PAP12	ARR11	ARR-B	AGAT
	ARR14	ARR-B	AGAT
	GLK 1	GARP	CCAATC
